# Supplementary material for: Structural insight into Okazaki fragment maturation mediated by PCNA-bound FEN1 and RNaseH2
Source: EMBO J. 2024 Nov 22;44(2):484–504. doi: 10.1038/s44318-024-00296-x (PMC11731006; doi:10.1038/s44318-024-00296-x)
Supplement: Supplementary file 1 — Appendix [file 44318_2024_296_MOESM1_ESM.pdf]

Appendix

Structural insight into the Okazaki fragment maturation by FEN1 and RNaseH2

Contents	Pages
Appendix Figure S1	2
Appendix Figure S2	3
Appendix Figure S3	4-5
Appendix Figure S4	6
Appendix Figure S5	7
Appendix Figure S6	8
Appendix Figure S7	9-10
Appendix Figure S8	11
Appendix Table S1	12
Appendix Table S2	13

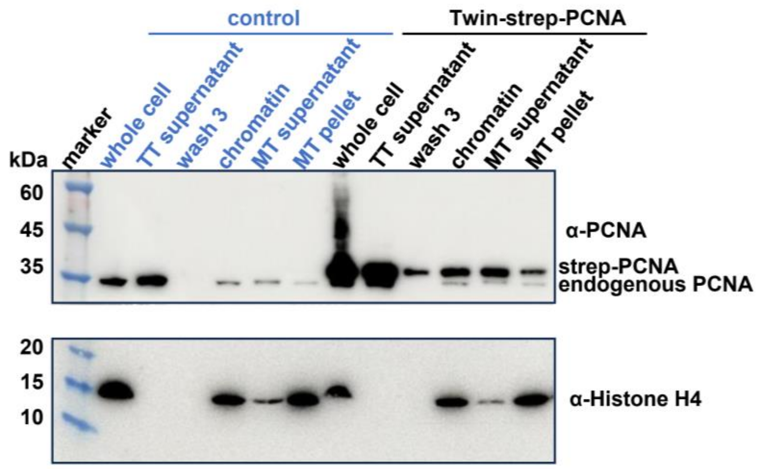

Appendix Figure S1. The extent of PCNA overexpression in HEK293 cells.

First, the cells were resuspended (whole cell) and lysed thoroughly with Triton-X100 treatment (TT). After lysis, the pellet obtained by centrifugation was washed using the resuspension buffer three times (wash1-3) to obtain the crude chromatin fractions (chromatin). Then, chromatin fractions were solubized by 5 kU/mL MNase treatment (MT) and clarified using centrifugation. For western blot analysis, commercially available mono-antibodies against PCNA (Proteintech, 60097-1-Ig) and histone H4 (Beyotime, AF2581) were used. Control, HEK293 cells; Twin-strep-PCNA, HEK293 cells transformed with plasmids encoding tagged PCNA.

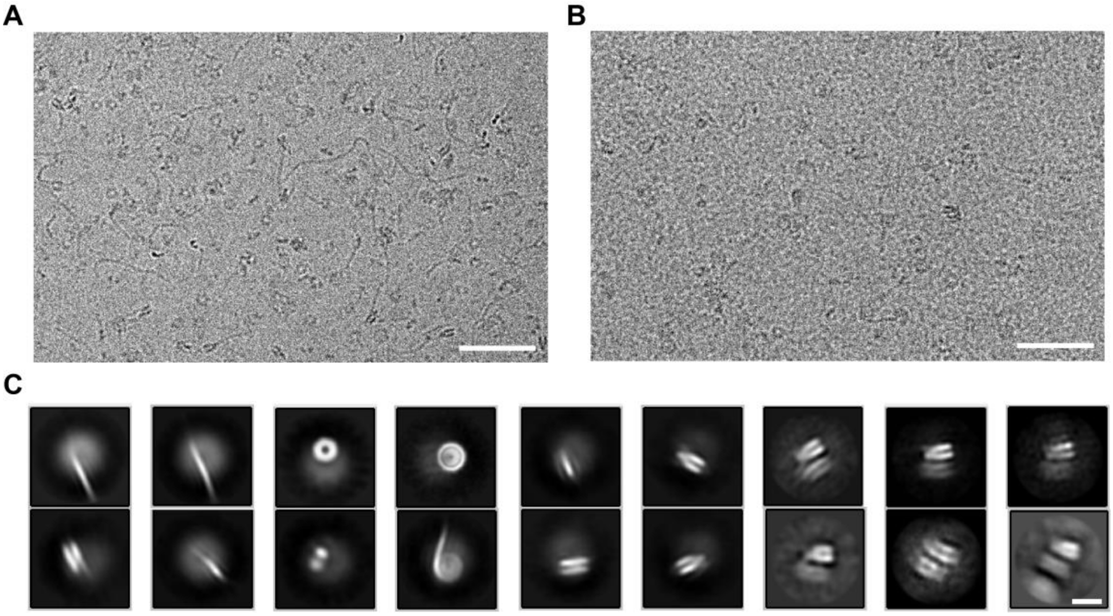

Appendix Figure S2. Structural profiling of endogenous PCNA-containing complexes.

(A-B) Representative cryo-EM particles prepared from Fractions 17-20 of the glycerol density gradient centrifugation. The grids used for panel a and b were coated with thin holey carbon film and thin continuous carbon film, respectively. Scale bar, 50 nm. **(C)** Representative average images from the 2D classification. Scale bar, 10 nm.

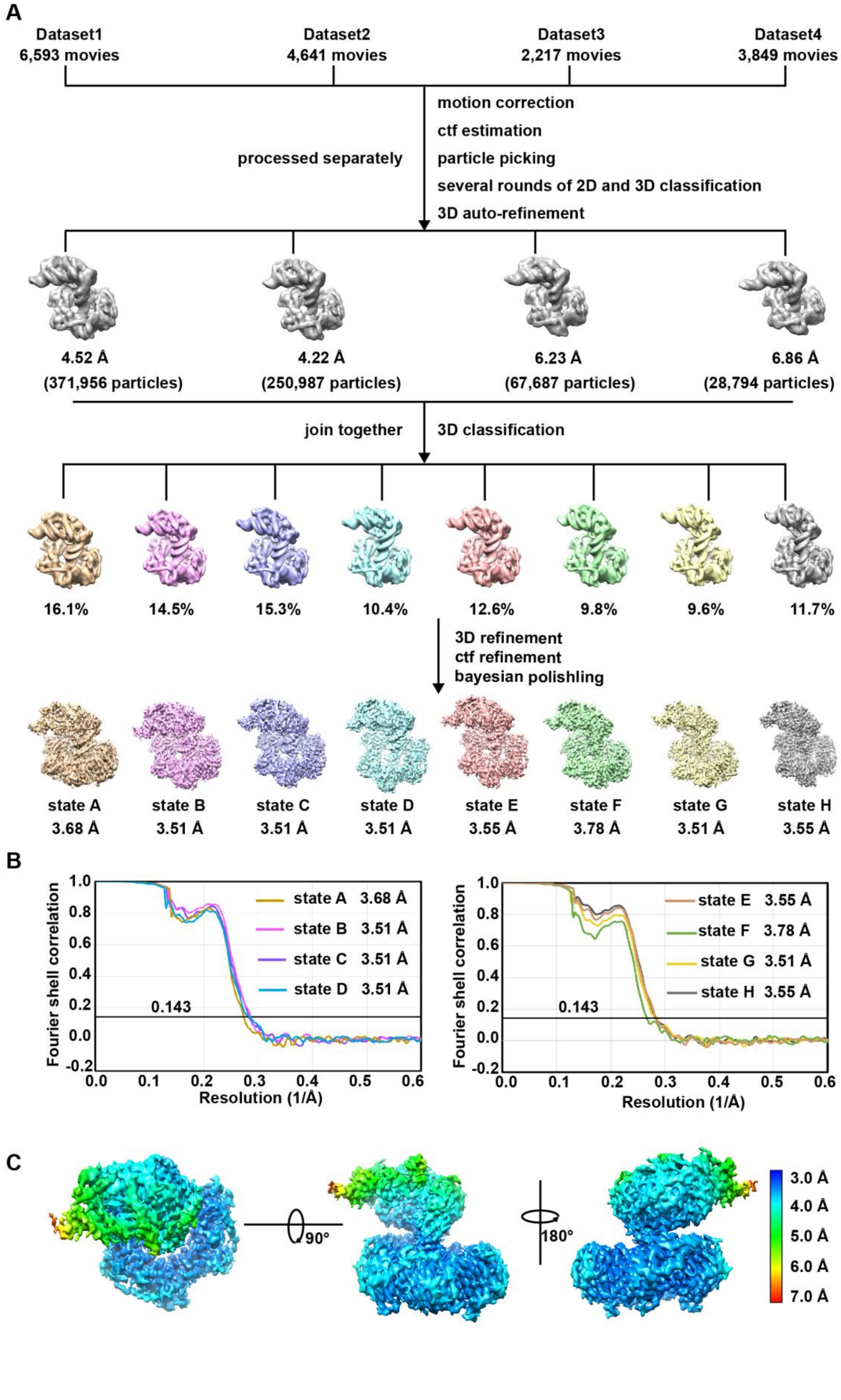
A
Dataset1 6,593 movies
Dataset2 4,641 movies
Dataset3 2,217 movies
Dataset4 3,849 movies
processed separately
motion correction
ctf estimation
particle picking
several rounds of 2D and 3D classification
3D auto-refinement
4.52 Å (371,956 particles)
4.22 Å (250,987 particles)
6.23 Å (67,687 particles)
6.86 Å (28,794 particles)
join together
3D classification
16.1%
14.5%
15.3%
10.4%
12.6%
9.8%
9.6%
11.7%
3D refinement
ctf refinement
bayesian polishling
state A 3.68 Å
state B 3.51 Å
state C 3.51 Å
state D 3.51 Å
state E 3.55 Å
state F 3.78 Å
state G 3.51 Å
state H 3.55 Å
B
Fourier shell correlation
Resolution (1/Å)
0.143
state A 3.68 Å
state B 3.51 Å
state C 3.51 Å
state D 3.51 Å
state E 3.55 Å
state F 3.78 Å
state G 3.51 Å
state H 3.55 Å
C
90°
180°
3.0 Å
4.0 Å
5.0 Å
6.0 Å
7.0 Å

Appendix Figure S3. Data processing of the PCNA-FEN1 complex.

(A) Flow chart of the image processing of the PCNA-FEN1 complex. The four datasets were first processed separately and the particles of the PCNA-FEN1 complex were combined for 3D classification and refinement. **(B)** FSC curves of eight PCNA-FEN1 density maps. The final resolution was estimated using the gold-standard FSC 0.143 cutoff. **(C)** Local resolution map of the PCNA-FEN1 complex in state D using ResMap.

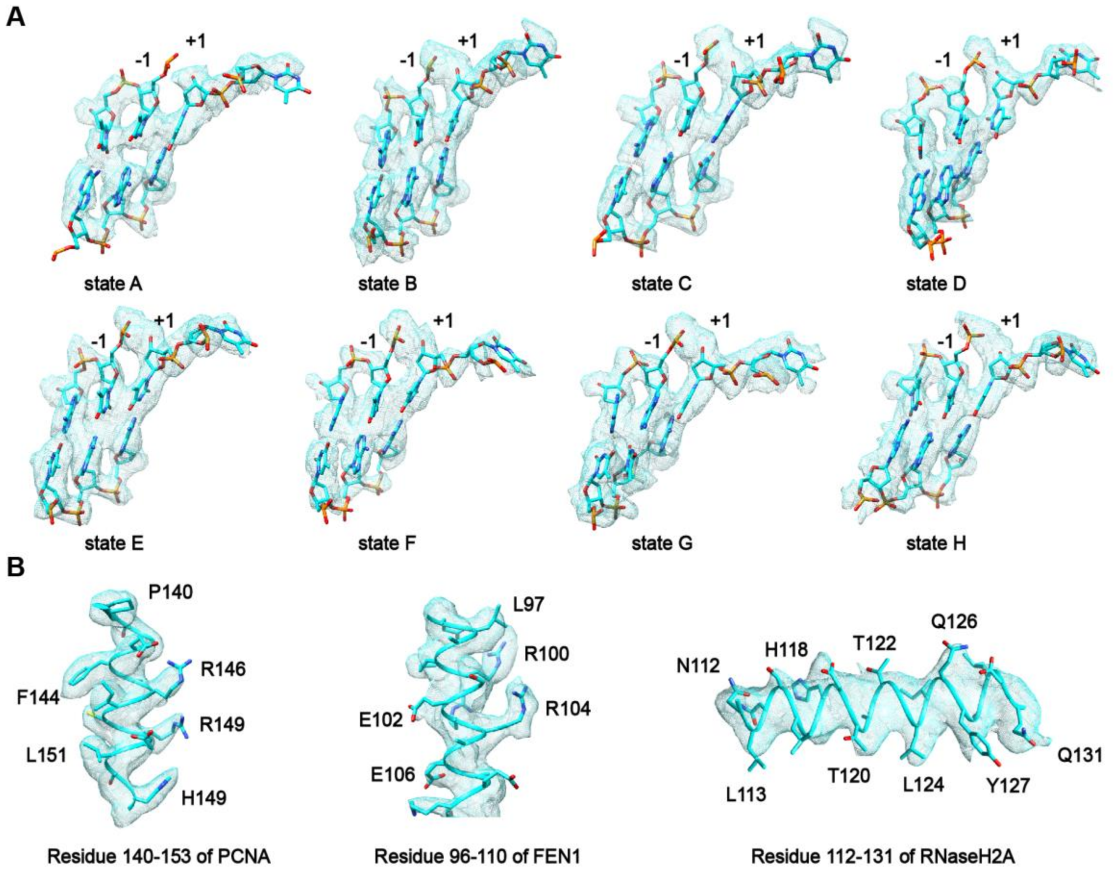

Appendix Figure S4. Local densities of the PCNA-FEN1 and PCNA-FEN1-RNaseH2 maps.

(A) Local density maps near the 5'-flap DNA of different states of the PCNA-FEN1 complex. The densities between the nucleotide at the +1 position and the nucleotide at the -1 position in all the maps show that the 5'-flap have already been cleaved. **(B)** Local densities of select regions from PCNA, FEN1 and RNaseH2 in the map of the PCNA-FEN1-RNaseH2 complex.

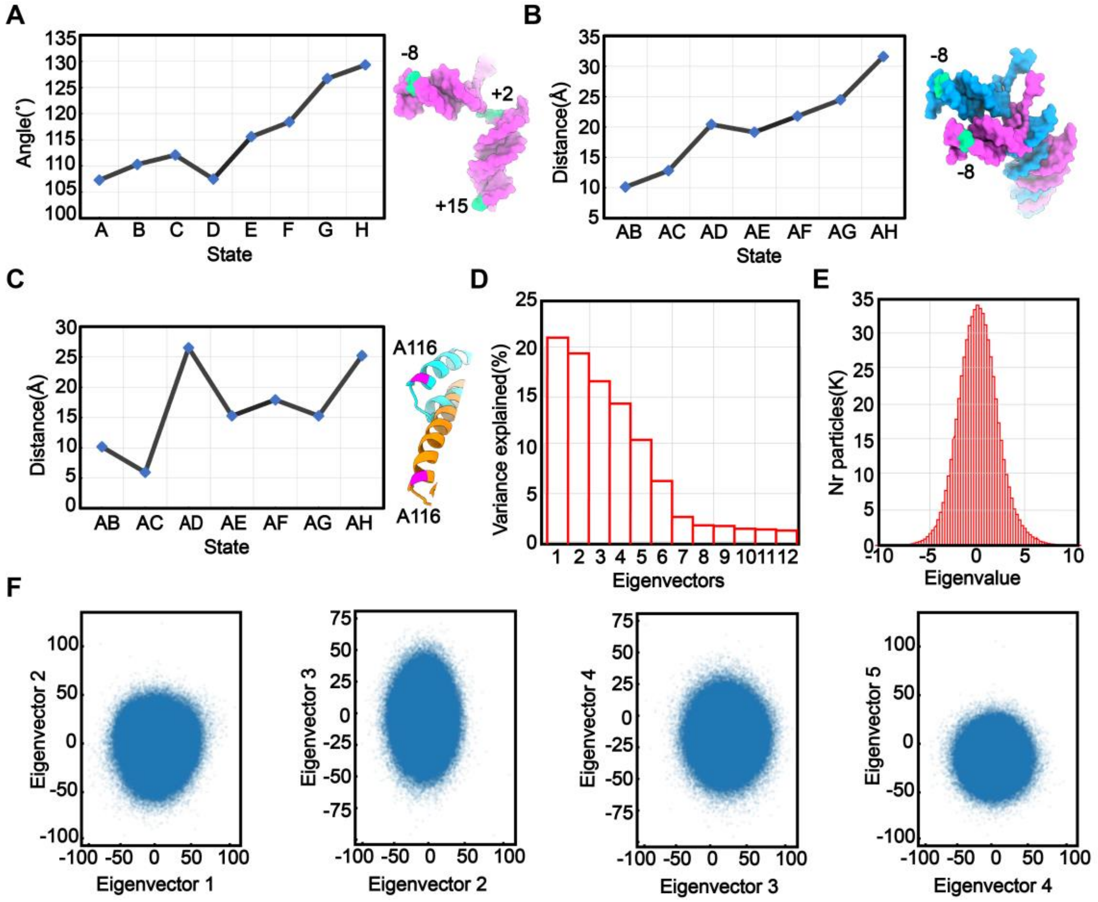

Appendix Figure S5. Continuous conformational transitions among structures of the PCNA-FEN1 complex.

(A) Measurement of the bending angle between the upstream and downstream dsDNA in different states. The P atom of the +2 nucleotide in the template strand is taken as the vertex and together with the P atom at -8 position of the downstream DNA and the P atom at +15 position of the upstream DNA define the angle between the upstream and downstream DNA. **(B)** Measurement of the displacement of the downstream DNA in states B-H, relative to the DNA in state A. The selected P of nucleotide is at -8 position of the downstream DNA. **(C)** Displacement of FEN1 in different states relative to that in state A. The distance measurement was done by calculating the distance of A116-Cα between two states. **(D)** 3D variance in the PCNA-FEN1 complex evaluated through multibody refinement. **(E)** The distribution of particles along the first eigenvector based on multibody refinement. **(F)** Scatter cloud plots of particles in eigenvector space according to the 3D variability analysis using cryo-SPARC.

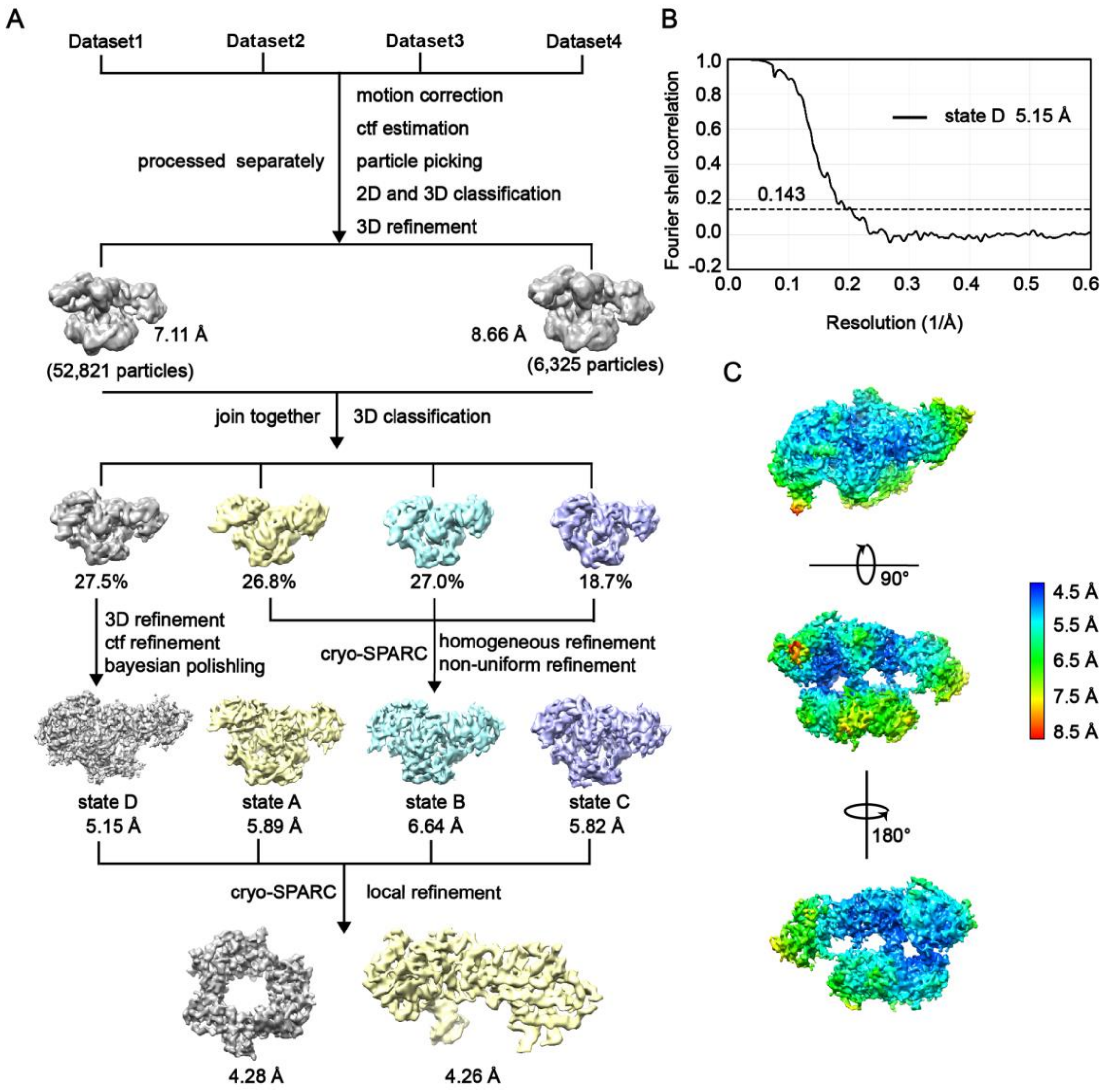

Appendix Figure S6. Data processing of the PCNA-FEN1-RNaseH2 complex.

(A) Flow chart of the data processing for the PCNA-FEN1-RNaseH2 complex. Particles of the PCNA-FEN1-RNaseH2 complex from different datasets were combined for 3D classification and refinement. **(B)** FSC curve of the PCNA-FEN1-RNaseH2 complex in state D. **(C)** Local resolution map of the final PCNA-FEN1-RNaseH2 structure in state D.

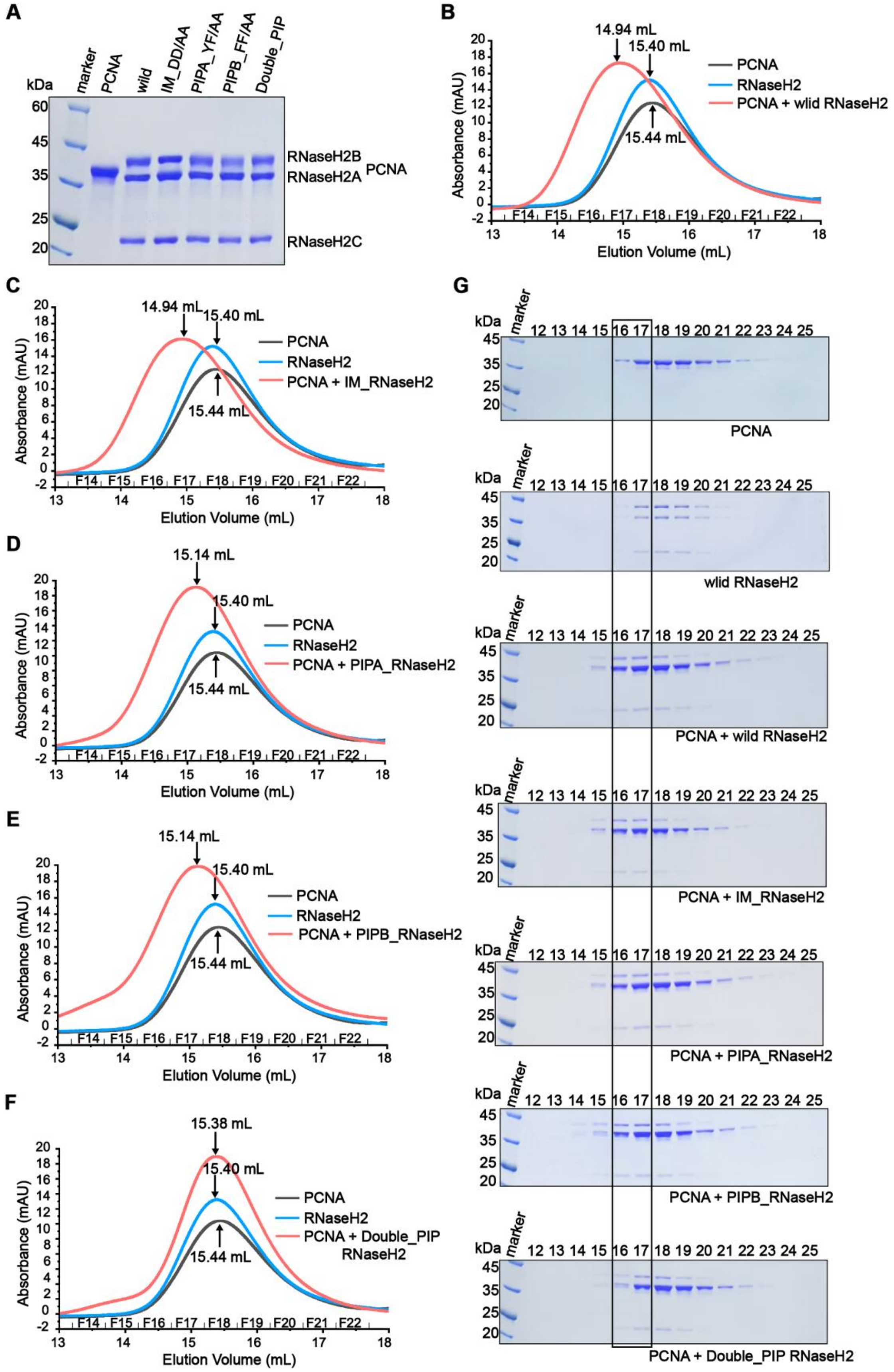

Appendix Figure S7. Both RNaseH2A PIP box and RNaseH2B PIP box contribute

to the binding to PCNA.

(A) Purified proteins of PCNA, wild-type and mutant RNaseH2 complex. **(B-F)** Size-exclusion chromatography of PCNA, RNaseH2 and their complexes. PCNA and RNaseH2 were incubated at a molar ratio of 1:1 for 30 min at RT and then loaded onto Superdex 200 for analysis. Wild, wild-type RNaseH2 complex; IM_DD/AA, mutant RNaseH2 complex, with a catalytical dead version of RNaseH2A; PIPA_YF/AA, mutant RNaseH2 complex, with a PIP box mutant version of RNaseH2A; PIPB_FF/AA, mutant RNaseH2 complex, with a PIP box mutant version of RNaseH2B; Double_PIP, mutant RNaseH2 complex, with both PIP boxes of RNaseH2A and RNaseH2B mutated. **(G)** Protein electrophoresis of selected fractions (12-25) in the panels B-F.

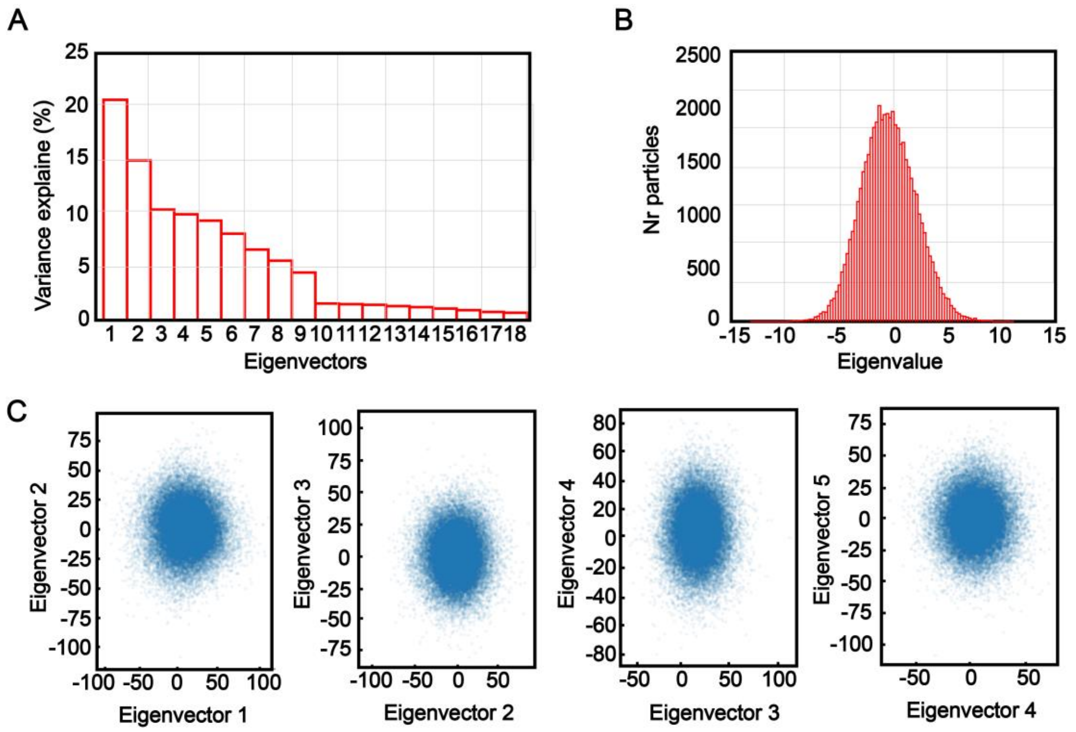

Appendix Figure S8. Continuous conformational transitions among structures of the PCNA-FEN1-RNaseH2 complex.

(A) Variance evaluated through multibody refinement. **(B)** The distribution of particles along the first eigenvector based on the results of multibody refinement using Relion. **(C)** Scatter cloud plots of particles in eigenvector space based on the results of 3D variability analysis using cryo-SPARC.

Appendix Table S1

Cryo-EM data collection, refinement and validation statistics

	State A PCNA-FEN1 (EMDB -39342) (PDB 8YJH)	State B PCNA-FEN1 (EMDB -39344) (PDB 8YJL)	State C PCNA-FEN1 (EMDB -39346) (PDB 8YJQ)	State D PCNA-FEN1 (EMDB -39347) (PDB 8YJR)	State E PCNA-FEN1 (EMDB -39348) (PDB 8YJS)	State F PCNA-FEN1 (EMDB -39350) (PDB 8YJU)	State G PCNA-FEN1 (EMDB -39351) (PDB 8YJV)	State H PCNA-FEN1 (EMDB -39352) (PDB 8YJW)	State D PCNA-FEN1-RNaseH2 (EMDB-39354) (PDB 8YJZ)
**Data collection and processing**									
Magnification					105,000 ×				
Voltage (kV)					300				
Electron exposure (e–/Å^2)					59.5				
Defocus range (μm)					-1.5 to -2.5				
Pixel size (Å)					0.83				
Symmetry imposed					C1				
Initial particle images (no.)					4,821,978				
Final particle images (no.)	98,129	89,413	94,546	64,551	78,778	59,825	59,594	72,922	13,906
Map resolution (Å)	3.68	3.51	3.51	3.51	3.55	3.78	3.51	3.55	5.15
FSC threshold	0.143	0.143	0.143	0.143	0.143	0.143	0.143	0.143	0.143
**Refinement**									
Map sharpening *B* factor (Å^2)	-105	-99	-81	-70	-82	-91	-93	-102	-147
Model composition									
Non-hydrogen atoms	9735	9853	9856	9962	9880	9939	9938	9946	14948
Protein residues	1115	1115	1115	1113	1111	1111	1111	1116	1744
Nucleotide residues	53	59	59	65	62	65	65	63	65
Ligands	0	0	0	0	0	0	0	0	0
R.m.s. deviations									
Bond lengths (Å)	0.005	0.005	0.005	0.005	0.006	0.005	0.007	0.006	0.005
Bond angles (°)	1.041	0.999	0.969	0.943	1.026	1.025	1.079	1.052	1.049
Validation									
MolProbity score	1.60	1.56	1.59	1.53	1.68	1.65	1.67	1.61	1.67
Clashscore	4.67	5.77	5.25	4.85	5.87	5.53	5.85	5.58	7.01
Poor rotamers (%)	0.00	0.00	0.00	0.00	0.00	0.00	0.00	0.00	0.00
Ramachandran plot									
Favored (%)	94.76	96.30	95.57	95.93	94.74	94.42	94.91	95.58	95.94
Allowed (%)	5.24	3.70	4.43	4.07	5.26	5.08	5.09	4.42	4.06
Disallowed (%)	0.00	0.00	0.00	0.00	0.00	0.00	0.00	0.00	0.00

Appendix Table S2

DNA used in Gel electrophoresis mobility shift assay

		sequence
5 bp	F	CGGTT
	R	AACCG
10 bp	F	CGGTTATAGG
	R	CCTATAACCG
15 bp	F	CGGTTATAGGTTCAC
	R	GTGAACCTATAACCG
20 bp	F	CGGTTATAGGTTCACGGGGT
	R	ACCCCGTGAACCTATAACCG
30 bp	F	CGGTTATAGGTTCACGGGGTTTTTAGATTT
	R	AAATCTAAAAACCCCGTGAACCTATAACCG
50 bp	F	AAAAACCCCGTGAACCTATAACCGTCTCTCTCTGCACTAAAAAATAGGG
	R	CCCTATTTTTTAGTGCAGAGAGAGACGGTTATAGGTTCACGGGGTTTTTA
