# Supplementary material for: Structural insight into Okazaki fragment maturation mediated by PCNA-bound FEN1 and RNaseH2
Source: EMBO J. 2024 Nov 22;44(2):484–504. doi: 10.1038/s44318-024-00296-x (PMC11731006; doi:10.1038/s44318-024-00296-x)
Supplement: Supplementary file 3 — Movie EV1 [file 44318_2024_296_MOESM3_ESM.zip › Movie EV1/Movie EV1 legend file.docx]

**Movie EV1**

Conformational changes in eight different states of PCNA-FEN1 structures with PCNA as the reference of alignment. This display proceeds in a clockwise progression along the downstream DNA terminus.
