# Supplementary material for: Structural insight into Okazaki fragment maturation mediated by PCNA-bound FEN1 and RNaseH2
Source: EMBO J. 2024 Nov 22;44(2):484–504. doi: 10.1038/s44318-024-00296-x (PMC11731006; doi:10.1038/s44318-024-00296-x)
Supplement: Supplementary file 4 — Movie EV2 [file 44318_2024_296_MOESM4_ESM.zip › Movie EV2/Movie EV2 legend file.docx]

**Movie EV2**

Conformational changes of the PCNA-FEN1 structure along the six dominant eigenvectors based on multibody refinement analysis.
