# Supplementary material for: Structural insight into Okazaki fragment maturation mediated by PCNA-bound FEN1 and RNaseH2
Source: EMBO J. 2024 Nov 22;44(2):484–504. doi: 10.1038/s44318-024-00296-x (PMC11731006; doi:10.1038/s44318-024-00296-x)
Supplement: Supplementary file 6 — Movie EV4 [file 44318_2024_296_MOESM6_ESM.zip › Movie EV4/Movie EV4 legend file.docx]

**Movie EV4**

Transition of different PCNA-FEN1-RNaseH2 functional states with PCNA as the reference of alignment.
