# Supplementary material for: Structural insight into Okazaki fragment maturation mediated by PCNA-bound FEN1 and RNaseH2
Source: EMBO J. 2024 Nov 22;44(2):484–504. doi: 10.1038/s44318-024-00296-x (PMC11731006; doi:10.1038/s44318-024-00296-x)
Supplement: Supplementary file 7 — Movie EV5 [file 44318_2024_296_MOESM7_ESM.zip › Movie EV5/Movie EV5 legend file.docx]

**Movie EV5**

Conformational changes of the PCNA-FEN1-RNaseH2 structure along the nine dominant eigenvectors based on multibody refinement analysis.
