# Supplementary material for: Structural insight into Okazaki fragment maturation mediated by PCNA-bound FEN1 and RNaseH2
Source: EMBO J. 2024 Nov 22;44(2):484–504. doi: 10.1038/s44318-024-00296-x (PMC11731006; doi:10.1038/s44318-024-00296-x)
Supplement: Supplementary file 8 — Movie EV6 [file 44318_2024_296_MOESM8_ESM.zip › Movie EV6/Movie EV6 legend file.docx]

**Movie EV6**

Detailed conformational changes in different space of the PCNA-FEN1-RNaseH2 structure along the first eigenvector based on multibody refinement analysis.
