# Supplementary figures and images for: Structural insight into Okazaki fragment maturation mediated by PCNA-bound FEN1 and RNaseH2
Source: EMBO J. 2024 Nov 22;44(2):484–504. doi: 10.1038/s44318-024-00296-x (PMC11731006; doi:10.1038/s44318-024-00296-x)

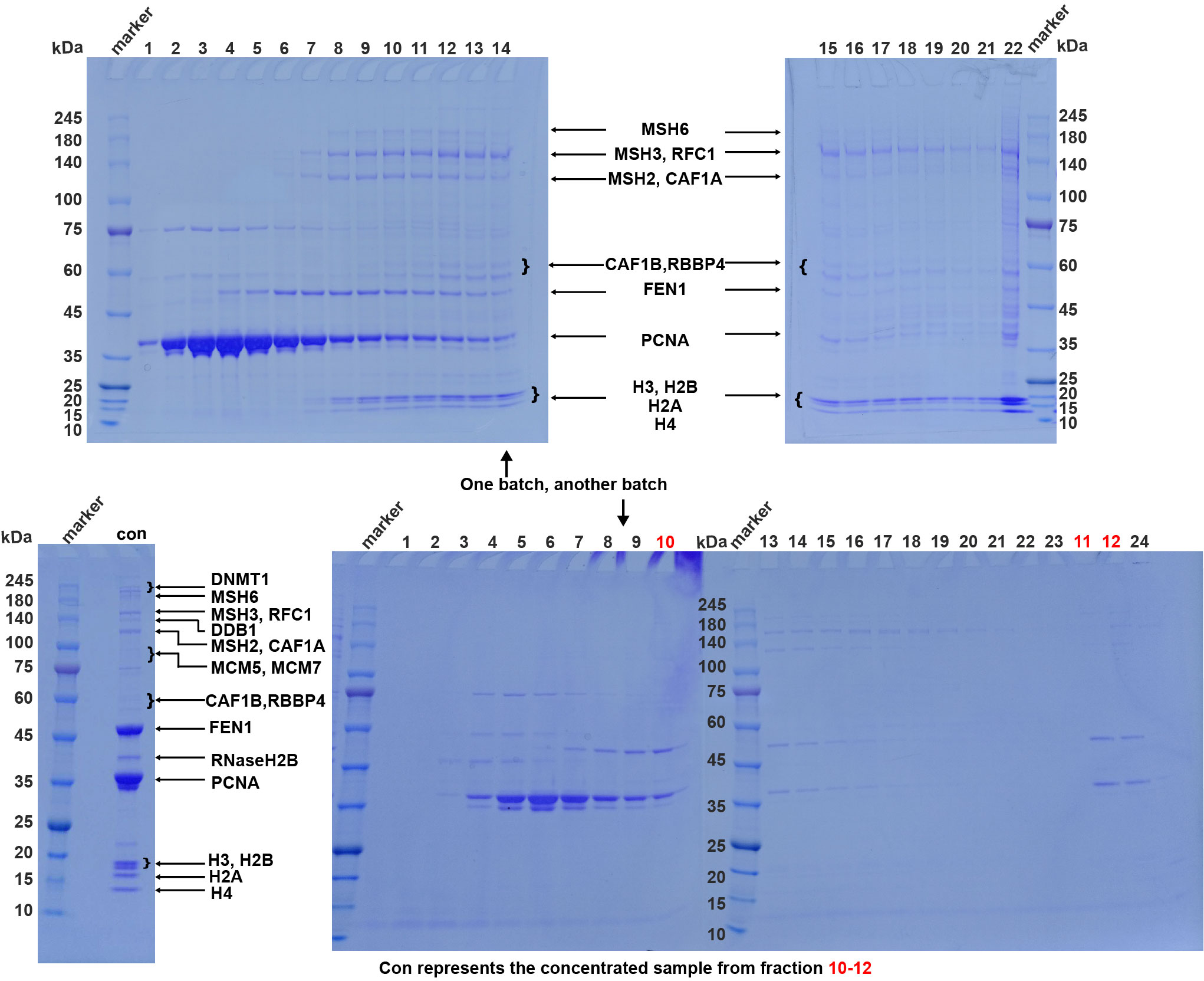

Supplement: Supplementary file 9 — Source data Fig. 1 [file 44318_2024_296_MOESM9_ESM.zip › SD figure 1/1B.jpg]

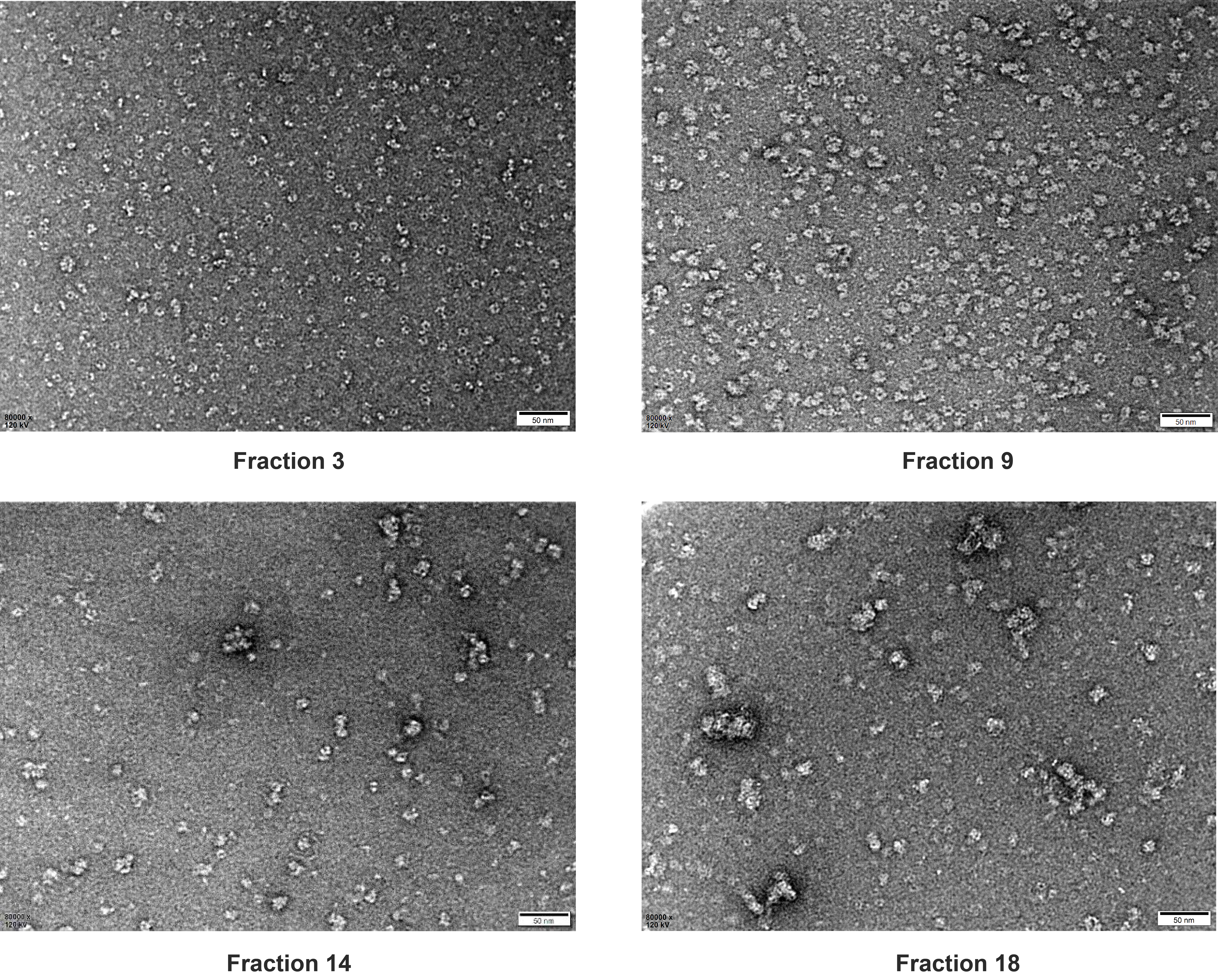

Supplement: Supplementary file 9 — Source data Fig. 1 [file 44318_2024_296_MOESM9_ESM.zip › SD figure 1/1C.jpg]

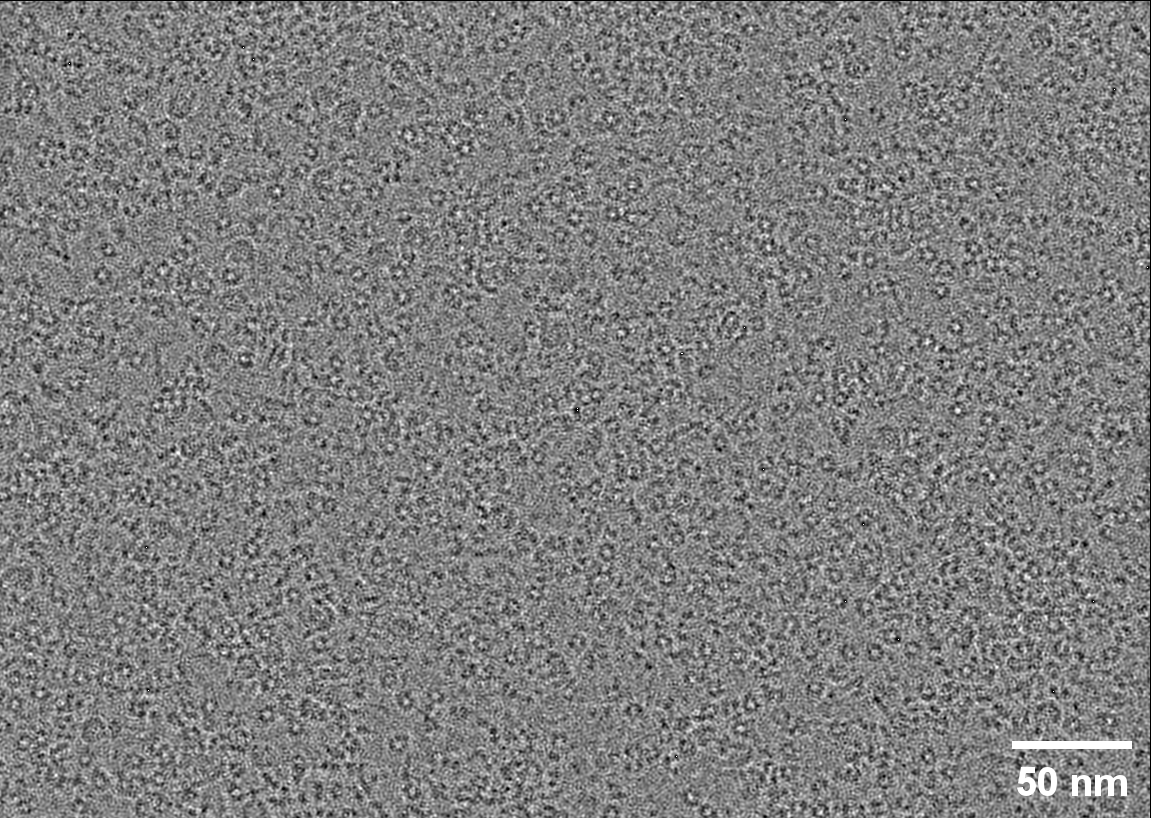

Supplement: Supplementary file 10 — Source data Fig. 2 [file 44318_2024_296_MOESM10_ESM.zip › SD figure 2/2A.tif]

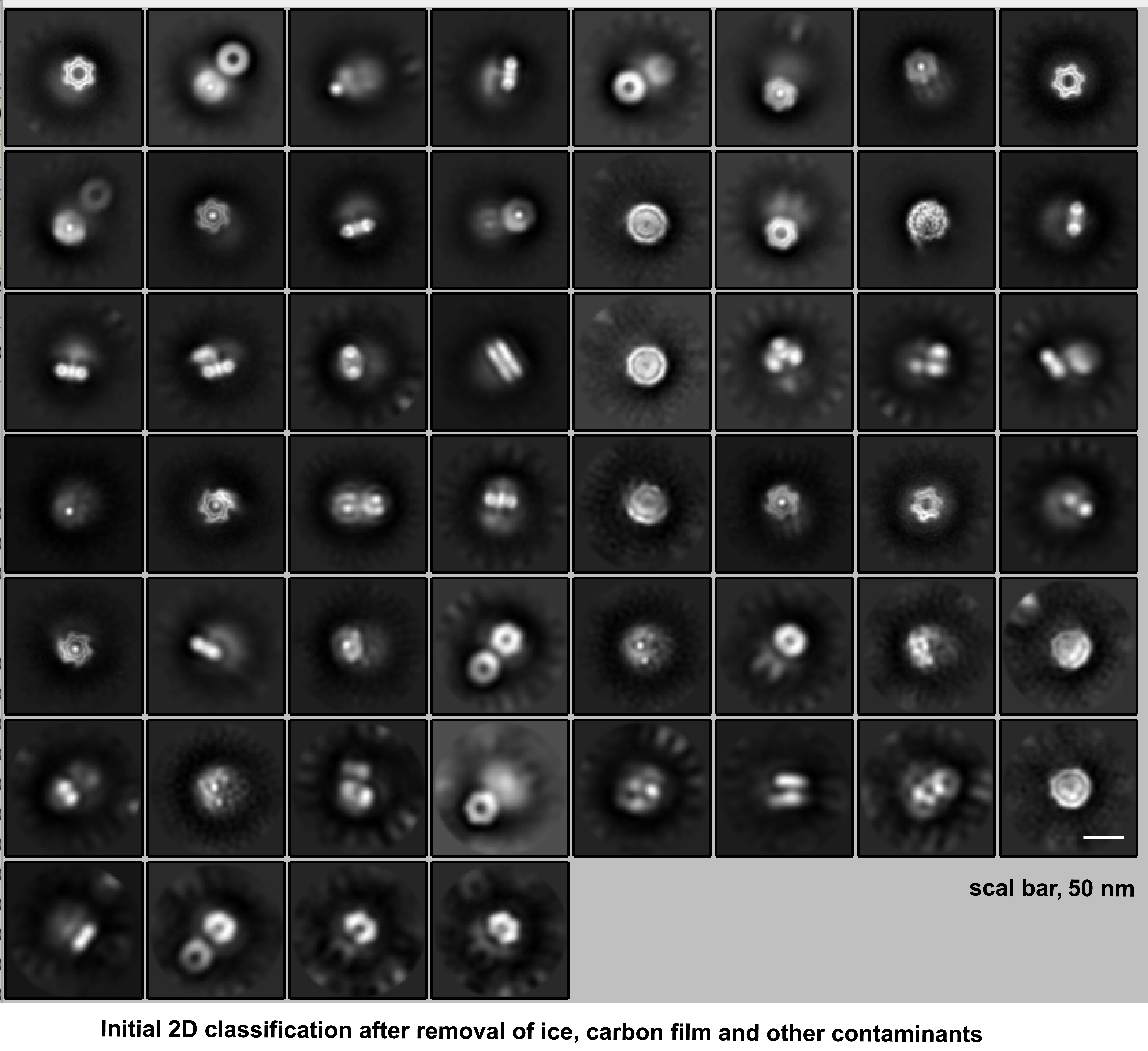

Supplement: Supplementary file 10 — Source data Fig. 2 [file 44318_2024_296_MOESM10_ESM.zip › SD figure 2/2B.jpg]

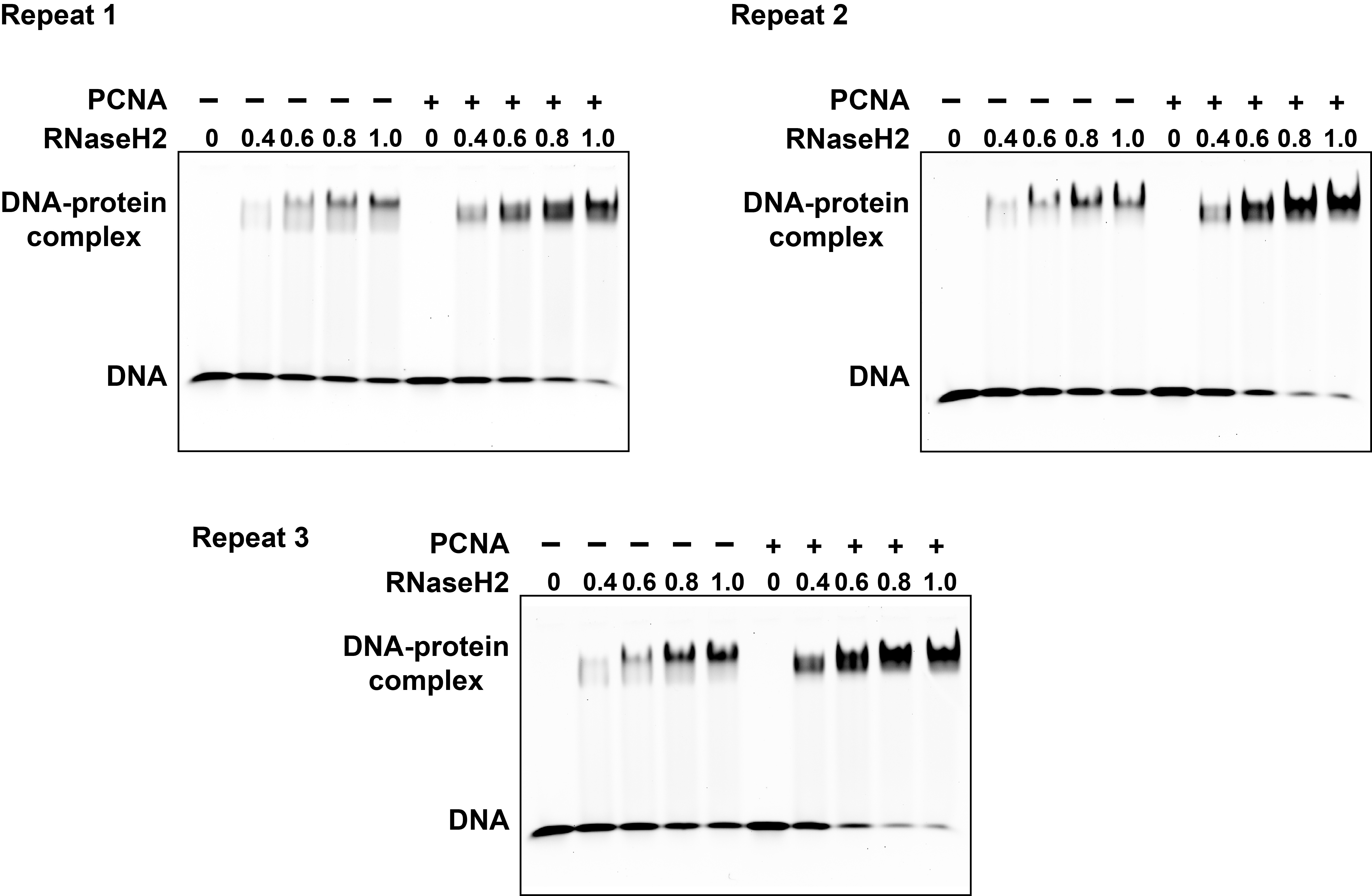

Supplement: Supplementary file 11 — Figure EV4C Source Data [file 44318_2024_296_MOESM11_ESM.zip › SD figure EV4C/Figure EV4C.jpg]
