# Supplementary material for: Structural insight into Okazaki fragment maturation mediated by PCNA-bound FEN1 and RNaseH2
Source: EMBO J. 2024 Nov 22;44(2):484–504. doi: 10.1038/s44318-024-00296-x (PMC11731006; doi:10.1038/s44318-024-00296-x)
Supplement: Supplementary file 12 — Expanded View Figures [file 44318_2024_296_MOESM12_ESM.pdf]

# Expanded View Figures

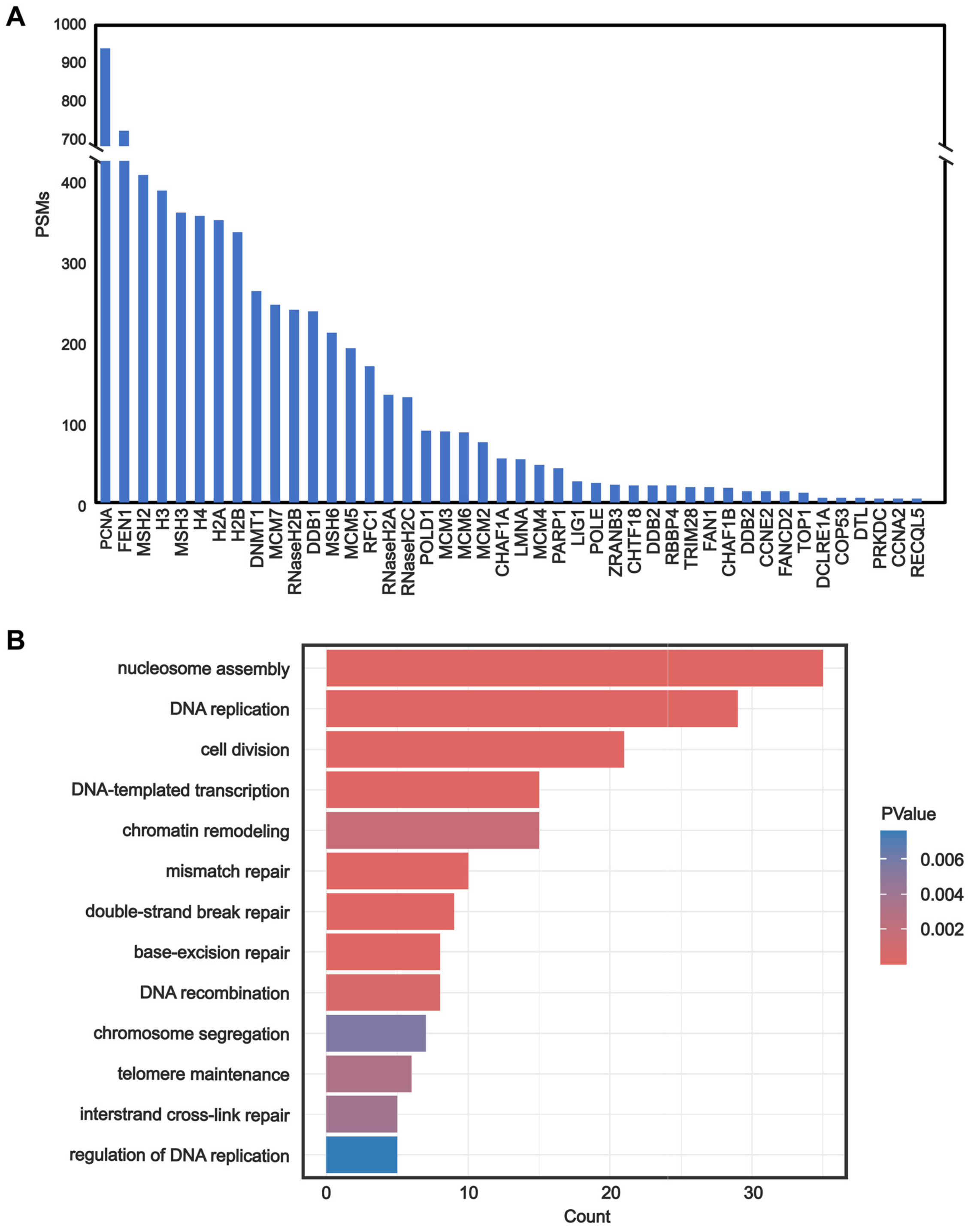


**Figure EV1. Mass spectrometric analysis of the obtained endogenous PCNA-containing complexes.**

(**A**) A list of representative proteins related to PCNA identified in our samples by mass spectrometry. Candidate proteins were selected according to the BioGRID (Oughtred et al, 2021) database, and only proteins with the total number of identified peptide sequences (PSMs) greater than or equal to 5 were selected. (**B**) Gene ontology analysis of proteins identified by mass spectrometry according to the biological process. A total of 333 identified proteins were subjected to GO analysis. The *P*-values were calculated using hypergeometric distribution tests.

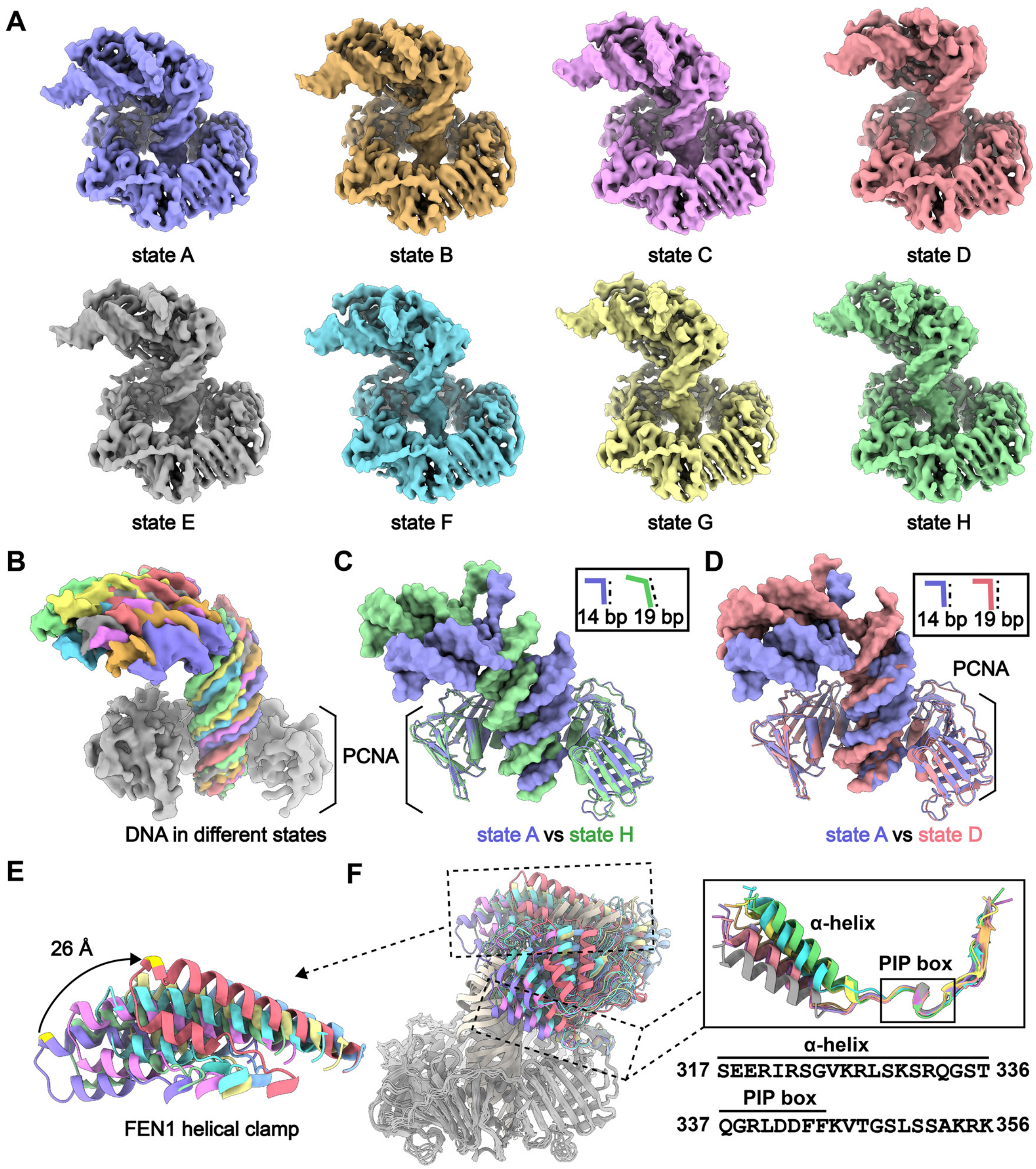


**Figure EV2. Different states of the PCNA-FEN1 complex.**

(**A**) Density maps of the eight conformational states. (**B**) Superimposition of the DNA in different states, with PCNA used as the reference of alignment. (**C**) Structural comparison of the DNA between state A and state H. The lengths of the upstream dsDNA in state A and state H are 14 bp and 19 bp, respectively. The bending angles of the DNA are significantly different between state A and state H. (**D**) Structural comparison of the DNA between state A and state D. The length of the upstream dsDNA in state D is 19 bp. The bending angles of the DNA in state A and state D are similar. (**E**) The positional change of the FEN1 helical clamp in different states, with PCNA used as reference of alignment. The distance between state A and state D is 26 Å measured by Cα of A116. (**F**) The C-terminus of FEN1 in different states. The conformation of the α-helix upstream of the PIP box has much greater changes than the PIP box.

 © The Author(s)

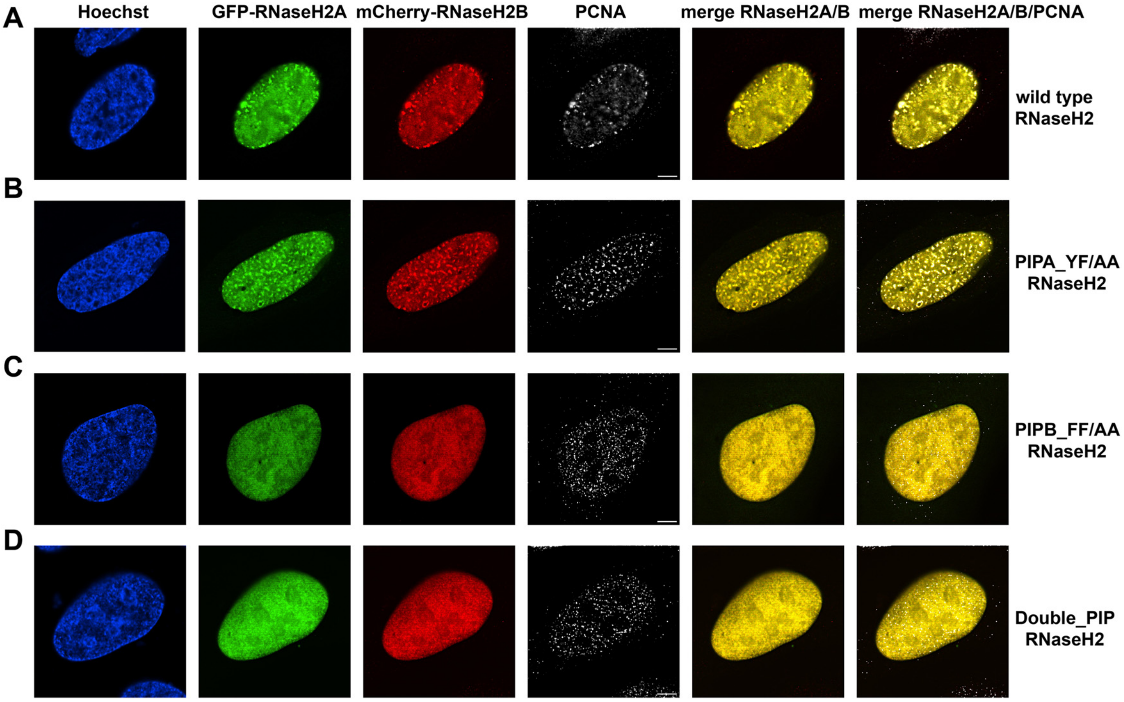


**Figure EV3. RNaseH2 depends on the PIP box of RNaseH2B to colocalize with PCNA.**

(**A**) GFP-RNaseH2A, mCherry-RNaseH2B colocalize with PCNA in U2OS cells. (**B**) Mutation of the PIP box in RNaseH2A does not affect its colocalization with PCNA. (**C**, **D**) GFP-RNaseH2A and mCherry-RNaseH2B distribute evenly in nuclei upon the mutation of RNaseH2B PIP box or double PIP box mutations in both RNaseH2A and RNaseH2B. PCNA was stained by immune-fluorescence with an Alexa fluor 647 secondary antibody. Scal bar, 5 μm.

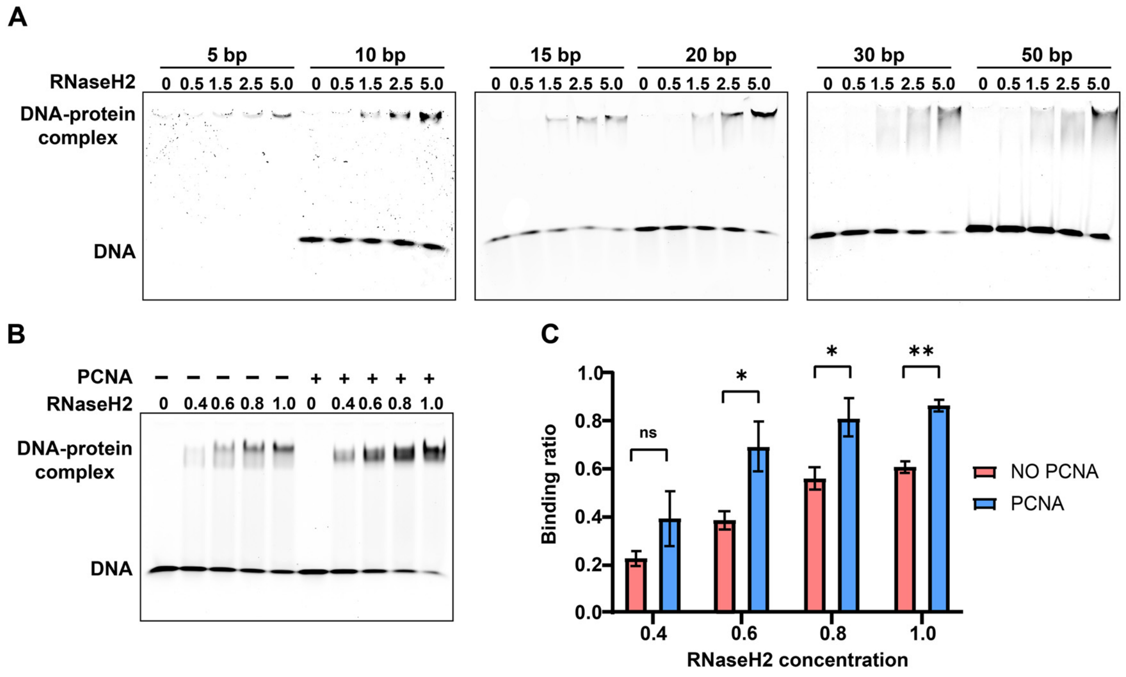


**Figure EV4. RNaseH2 binds strongly to the dsDNA of varying length.**

(**A**) RNaseH2 binds to the dsDNA in different length. The label-free dsDNA (2.5 μM) was incubated with RNaseH2 at different concentrations (0.5, 1.5, 2.5, 5 μM) for 30 min at RT and analyzed by 5% native TBE-PAGE gel. (**B**) PCNA promotes the binding of RNaseH2 to the dsDNA. 5′-FAM-labeled 20-bp dsDNA (100 nM) was incubated with RNaseH2 at different concentrations (0.4, 0.6, 0.8, 1.0 μM) in the presence or absence of PCNA (100 nM) for 30 min at RT and analyzed by 5% native TBE-PAGE gel. (**C**) Quantitative analysis of the binding of RNaseH2 to the dsDNA. The DNA-protein complexes in (**B**) were quantified using ImageJ, followed by statistical analysis using GraphPadPrism ($n = 3$ biological replicates). ns, not significant, *$p < 0.05$, **$p < 0.001$, multiple paired t tests. *P* values of PCNA vs NO PCNA: 0.092 (0.4 μM RNaseH2), 0.025 (0.6 μM RNaseH2), 0.010 (0.8 μM RNaseH2), 0.003 (1.0 μM RNaseH2). Error bars based on standard deviation (s.d.). Source data are available online for this figure.

 © The Author(s)

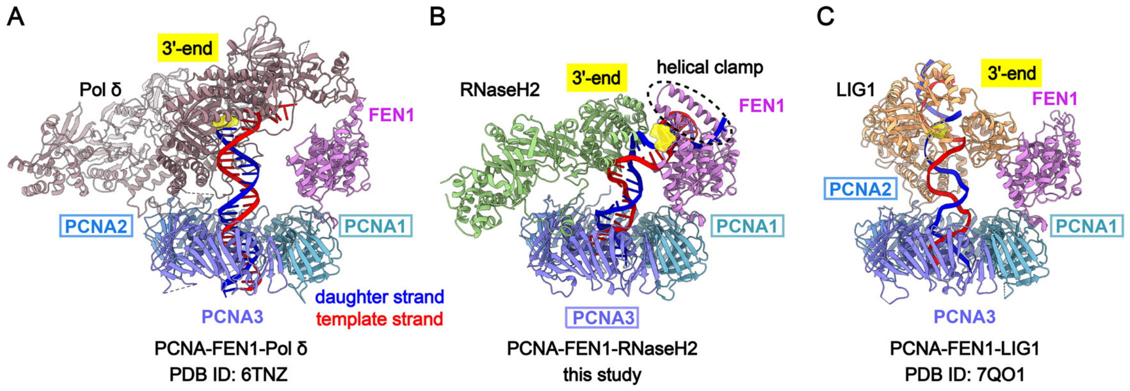


**Figure EV5. Structural comparison of the PCNA-FEN1-Pol δ complex, the PCNA-FEN1-RNaseH2 complex, and the PCNA-FEN1-LIG1 complex.**

(**A**) The structure of the in vitro assembled PCNA-FEN1-Pol δ complex (PDB ID: 6TNZ) (Lancey et al, 2020). The PIP boxes of FEN1 and Pol δ docks onto the first and the second PCNA monomers (PCNA1 and PCNA2), respectively. The two strands of the DNA are separately colored. The 3′-end of the daughter strand is colored yellow and shown in surface representation. (**B**) The structure of the endogenous PCNA-FEN1-RNaseH2 complex. FEN1 and RNaseH2 bind to the first and the third PCNA monomers (PCNA1 and PCNA3), respectively. (**C**) The structure of the in vitro assembled PCNA-FEN1-LIG1 complex (PDB ID: 7QO1) (Blair et al, 2022). FEN1 and LIG1 bind to the first and the second PCNA monomers (PCNA1 and PCNA2), respectively. The three structures are displayed with PCNA monomer (PCNA1) as the reference of alignment.
